# Use and perceived helpfulness of smoking cessation methods: results from a population survey of recent quitters

**DOI:** 10.1186/1471-2458-11-592

**Published:** 2011-07-27

**Authors:** Wai Tak Hung, Sally M Dunlop, Donna Perez, Trish Cotter

**Affiliations:** 1 Monitoring, Evaluation and Research Unit, Cancer Institute NSW, Level 9, 8 Central Avenue, Australian Technology Park, Eveleigh NSW 2015, Australia; 2 Cancer Prevention Division, Cancer Institute NSW, Level 9, 8 Central Avenue, Australian Technology Park, Eveleigh NSW 2015, Australia; 3 Sydney School of Public Health, The University of Sydney, Edward Ford Building, NSW 2006, Australia

## Abstract

**Background:**

Increasing rates of smoking cessation is one of the most effective measures available to improve population health. To advance the goal of increasing successful cessation at the population level, it is imperative that we understand more about smokers' use of cessation methods, as well as the helpfulness of those methods in real-world experiences of quitting. In this survey of recent quitters, we simultaneously examined rates of use and perceived helpfulness of various cessation methods.

**Methods:**

Recent quitters (within 12 months; n = 1097) completed a telephone survey including questions relating to 13 cessation methods. Indices of use and perceived helpfulness for each method were plotted in a quadrant analysis. Socio-demographic differences were explored using bivariate and multivariate analyses.

**Results:**

From the quadrant analysis, cold turkey, NRT and gradual reduction before quitting had high use and helpfulness; GP advice had high use and lower helpfulness. Prescribed medication and online programs had low use but high helpfulness. Remaining methods had low use and helpfulness. Younger quitters were more likely to use unassisted methods such as cold turkey; older or less educated quitters were more likely to use assisted methods such as prescribed medication or advice from a general practitioner.

**Conclusions:**

The majority of recent quitters quit cold turkey or cut down before quitting, and reported that these methods were helpful. Efforts to influence population smoking prevalence should attempt to provide support and motivation for smokers choosing these methods, in addition to assessing the effectiveness and accessibility of other methods for smokers who need or choose them.

## Background

Cigarette smoking remains the leading preventable cause of death and disease in the developed world [1,2], and increasing the number of smokers successfully quitting is one of the most effective measures available to improve population health. Cessation activity among smokers is relatively common; more than 40% of current U.S. smokers report having made a serious attempt to quit in the past 12 months [3] and in the Australian state of New South Wales (NSW) less than 20% of smokers have never tried to quit [4]. However, only about 3% - 5% of smokers maintain abstinence up to one year after quitting [5]. To advance the goal of increasing successful cessation at the population level, it is imperative that we understand *how *smokers quit, one aspect of which is the use and helpfulness of various cessation methods and aids.

Over the last decade, numerous studies have documented the proportion of smokers using various cessation methods. In the U.S., it is estimated that around one quarter of smokers use pharmacologic treatments such as nicotine replacement therapy (NRT) and bupropion when attempting to quit [3,6,7]. The use of behavioural treatments is estimated to be under 8% [3,7]. Among behavioural treatments, self-help material is the most commonly used, but others include individual, group and telephone counselling [3]. In NSW, the most common aid used in quit attempts is NRT (approximately 33%), followed by bupropion (13.2%), with very small proportions of smokers reporting that they used behavioural aids such as telephone helplines [4,8]. Despite the increasing availability and marketing of pharmacological and behavioural interventions, population studies consistently show that the largest proportion of smokers who permanently quit smoking do so without any form of assistance [3,6,8-12]. That is, the most common method used by people who have successfully stopped smoking remains unassisted cessation (cold turkey or reducing before quitting).

When evaluating cessation methods, it is important to consider not just how *often *a cessation method is used, but also how *helpful *it is achieving smoking abstinence. Research investigating the effectiveness of various cessation methods and aids has fallen primarily into two categories - randomized controlled trials and retrospective population surveys. Controlled trials have generally demonstrated the efficacy of behavioural and pharmacological cessation interventions [13]. However, there has been a divergence between the findings from these trials and the results of retrospective population surveys in which smokers who report using behavioural or pharmacologic treatment are *less *likely to be successful in quitting [3,8,11,14]. Only one recent survey has indicated greater quitting success in individuals who sought assistance than those not seeking assistance [6]. Some researchers have suggested that the inconsistency between the controlled studies and population surveys may be due to selection bias, with heavier or more addicted smokers being more likely to use cessation programs [3,6,7,11], and also being the most likely to relapse [15].

In the current study, using reports from recent quitters, we take into account both the reported use and perceived helpfulness of different cessation methods. In doing so, we can identify methods which have high impact - those which are used frequently and rated as helpful - as well as those which are rated as helpful, but used less frequently. The latter category could be a target for increased promotion. Conversely, identifying methods which have a low helpfulness rating could inform policy decisions and research priorities. We explore differences in use and perceived helpfulness of cessation methods by quitters who vary by socio-demographic characteristics, as well as by length of abstinence.

Throughout the study period (2007-2009), a range of quitting methods was available to smokers in Australia. A free national telephone cessation helpline (the Quitline) exists and is advertised on all cigarette packets and in media campaigns. NRT has been available over-the-counter since 1997. Bupropion (Zyban^®^) has been available since 2000 by prescription (with government subsidy since early 2001) and prescribed varenicline (Champix^®^) has been available with subsidy since January 2008. Additionally, a number of community-outreach groups provide counselling and support, and web-based support is increasingly available.

## Method

### Participants and Procedure

Data come from the Cancer Institute NSW's Tobacco Tracking Survey (CITTS). The CITTS is a telephone survey of adult smokers and recent quitters (aged 18 years and over) which monitors smoking-related cognitions, intentions and behaviours in the community, as well as responses to tobacco control policies and ongoing anti-smoking media campaigns. Households are recruited to the telephone survey using random digit dialling and participants are recruited using a random selection procedure. Fifty interviews per week are conducted across most weeks of the year; data for this study was collected between April 2007 and December 2009. An overall response rate of 27.4% was achieved for this period (American Association for Public Opinion Research Response Rate #4 [16]), with a total sample of 7,085 adults (both smokers and recent quitters). Weights were applied to adjust for gender, age and region, according to the NSW population [17]. Analyses for this study are limited to individuals who reported having quit smoking within the last 12 months (recent quitters, *n *= 1097).

### Measures

#### Cessation methods

Respondents were asked 'Did you use any of the following to help you quit smoking?'. The cessation methods that respondents were queried about were: NRT (including gum, lozenges, patches or inhalers), prescribed medication, the Quitline, online quit smoking information or programs, other 'how to quit' or 'self-help' materials, cold turkey, cutting down on the amount smoked before quitting (gradual reduction), changing to 'light' cigarettes, advice from dentist, advice from a general practitioner (GP), advice from a pharmacist, advice from another health professional, and natural or alternative therapies (e.g. hypnotherapy, acupuncture, laser therapy). Given that many smokers use one or more methods when quitting [3,8], respondents could nominate multiple methods. If they answered that they used a cessation method, they were asked whether it had helped them 'a great deal', 'somewhat' or 'not at all' (dichotomised into 'at least somewhat' vs. 'not at all').

Respondents also had the opportunity to report any other aid or method they used or to state that 'none of the above' helped them. Other methods were recorded verbatim, and recoded back into the original list if appropriate. Respondents who answered that they used 'none of the above' and did not specify any other methods (*n *= 28), or answered 'don't know' (*n *= 1), were excluded from analyses (resulting weighted sample, *n *= 1068).

#### Smoking and Demographic Characteristics

Demographic items measuring age, gender and education level (grouped into high school or less vs some or completed tertiary education) were included in the survey. Respondents were asked how long ago they quit smoking ('in the last two weeks', 'in the last month', 'in the last six months', or 'in the last year'). Postcodes were used with the Socio-Economic Indices for Areas [SEIFA; [18]] to indicate socio-economic status (quintiles four and five classified as low SES, and quintiles one to three as moderate-high SES).

### Statistical Analyses

First, we used a quadrant analysis to simultaneously examine the use and perceived helpfulness of each method [19]. In the quadrant analysis, an index of use for each method was plotted along the x-axis, whilst an index of perceived helpfulness for each method was plotted on the y-axis. First, the proportion of respondents who used a particular cessation method was calculated (*x*_*i*_). Next, the overall proportion of use of any cessation method (*X*) was obtained by calculating the weighted average of all *x*_*i *_using the corresponding sample size (*n*_*i*_) as the weight. Using a weighted average allowed for any method with a larger base sample size to have a greater contribution to the overall proportion of use. An index of use for each method was calculated by dividing *x*_*i *_by *X *and multiplying by 100, such that a score of 100 was the average. The index of perceived helpfulness was created similarly.

The four quadrants were thus defined: Quadrant I has methods with indices of use and perceived helpfulness both greater than or equal to 100 (high use and high helpfulness); Quadrant II has methods that have an index of use greater than or equal to 100 but an index of helpfulness less than 100 (high use and low helpfulness); Quadrant III has methods that have an index of use less than 100 but an index of helpfulness greater than or equal to 100 (low use but high helpfulness); Quadrant IV has methods with indices of use and helpfulness both less than 100 (low use and low helpfulness).

Next, we conducted bivariate analyses to determine associations between individual characteristics and use or perceived helpfulness of cessation methods, using chi-squared tests or Fisher's Exact test (for two-by-two contingency tables), and assessing linear trend for age. Finally, for each of the most frequently used methods which fell into Quadrants I, II and III, separate multiple logistic regression analyses were used to predict their use and perceived helpfulness. Following Hosmer and Lemeshow's recommendations for logistic regression with a large potential set of predictors [20], individual characteristics that had at least some degree of association with the outcome variable (*p *< 0.2) were entered into a backward stepwise logistic regression to predict the outcomes of interest. Only data from respondents who had no missing data on the variables of interest were included in each analysis; missing data on demographic variables reduced sample sizes slightly below the total number of cases available (see *n*s in tables). All analyses were performed using SPSS version 15 [21].

## Results

### Sample Characteristics

The final sample was 54% female. Twenty-four percent of the sample was aged 18-30 years, 41% were 31-50 years, and 35% were over 50 years. Sixty-five percent of these recent quitters had been abstinent less than six months (9% quit in the last 2 weeks; 12% in the last month; 44% more than one month ago but less than 6 months ago; 35% more than 6 months ago). Slightly fewer than half (46%) of the sample had high school or less education, and 36% were classified as low SES.

### Use and Perceived Helpfulness of Cessation Methods

**Figure 1:**
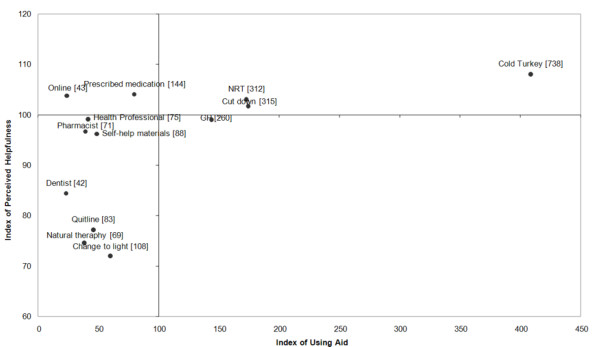
**Quadrant analysis of different quitting methods**. Note. Numbers in brackets are sample sizes for use of each method.

Sixty-six percent of recent quitters reported that they had used one or two of the cessation methods to help them quit, and 34% had used three or more. The most frequently cited cessation method was cold turkey, with 69% of respondents reporting that they used this method. The next two most common methods were gradual reduction and NRT (both 29%). Figure 1 shows the quadrant analysis: cold turkey, NRT and gradual reduction were in Quadrant I; GP advice was in Quadrant II; prescribed medication and online programs were in Quadrant III; and all remaining methods were in Quadrant IV.

### Bivariate Associations between Characteristics of Recent Quitters and Use and Perceived Helpfulness of Cessation Methods

The use of the individual cessation methods were analysed to investigate differences by respondent characteristics (see Table 1). Use of NRT, GP advice, health professional advice, natural therapy, and prescribed medication showed a significant positive trend of age, in that use was higher among older compared to younger respondents. In contrast, gradual reduction and cold turkey showed significant negative trends for age, in that use was most common among younger respondents. Chi-squared tests showed a significant age differences for use of dentist advice and the Quitline, with highest use among the middle age group (31-50 years). Use of an online program was significantly associated with gender; more females used an online program than males, though use for both genders was low.

**Table 1:** Recent quitters' use of cessation methods by socio-demographic and quitting characteristics

	USE OF METHODS (%)												
	Aided methods										Unaided methods		
	**NRT**	**Dentist**	**GP**	**Pharmacist**	**Health prof.**	**Natural therapy**	**Prescribed meds**	**Quitline**	**Self-help materials**	**Online**	**Change to light**	**Cut down**	**Cold turkey**
	
**Gender**													
Male (*n *= 488)	27.2	4.7	27.0	7.0	8.2	5.1	12.9	6.4	7.0	2.7*	9.8	31.1	69.9
Female (*n *= 580)	30.9	3.3	22.1	6.2	6.0	7.6	13.8	9.0	9.5	5.2	10.3	28.1	68.4
**Age (yrs)**													
18-30 (*n *= 254)	17.7***	1.6*	13.4***	4.3	2.8**	2.8*	5.9***	3.5*	8.7	4.7	9.8	34.6^a^	80.3***
31-50 (*n *= 436)	32.8	5.7	22.7	8.3	6.7	7.4	12.8	9.9	9.4	5.1	10.8	29.4	66.1
51+ (*n *= 377)	32.9	3.5	34.0	6.4	10.6	8.0	19.1	8.2	6.6	2.4	9.3	26.1	64.9
**Edu.**													
Low (*n *= 483)	30.2	4.1	30.0***	7.0	6.8	4.6*	17.2**	7.6	6.6	3.3	9.5	29.1	67.9
High (*n *= 573)	28.1	3.8	19.8	6.1	7.2	8.2	10.0	8.0	9.6	4.7	10.5	29.9	70.7
**SES**													
Mod-high (*n *= 657)	28.7	4.3	21.9*	5.6	7.3	6.7	11.1*	8.2	9.9*	4.4	10.0	28.0	72.5**
Low (*n *= 373)	30.8	3.2	28.7	8.0	7.2	6.2	16.9	7.3	5.6	2.7	10.2	31.9	63.3
**Quit duration**													
0-6 mths (*n *= 690)	31.4*	4.5	26.0	8.1**	6.2	6.2	16.2***	8.8	8.4	4.1	9.9	28.8	65.7**
7-12 mths (*n *= 378)	25.1	2.9	21.4	3.7	8.7	7.1	8.5	5.8	8.2	4.0	10.3	30.7	75.4

Note. Health prof = Health professional advice; Edu = Education (low = less than Year 12, high = some or completed tertiary); SES = socio-economic status; statistical tests of differences between groups are chi-square or Fisher's Exact test;***p < .001, **p < .01, *p < 0.05; ^a^chi-square test not significant but linear trend *p *< 0.05.

The use of NRT, pharmacist advice and prescribed medication were each significantly associated with quit duration; use was greater among respondents who had been abstinent six months or less compared to those abstinent more than 6 months. Conversely, cold turkey was used by a significantly larger proportion of quitters who had been abstinent for more than six months. Use of GP advice and prescribed medication was higher among respondents of low SES compared to those of moderate-high SES. However, use of self-help materials and cold turkey was higher among those of moderate-high SES compared to low SES. Use of GP advice and prescribed medication was also higher among respondents with high school or less education compared to those with tertiary education. Use of alternative therapies was higher among those with tertiary education.

We also considered differences in perceived helpfulness of each aid or method according to individual characteristics (see Table 2). For NRT and GP advice, there was a significant positive trend of age, in that a higher proportion of older respondents reported being helped by those methods compared to younger respondents. In contrast, changing to 'lights', calling the Quitline, and using online programs showed a significant negative trend of age, in that a higher proportion of younger respondents reported that these methods were helpful compared to older respondents. A higher proportion of low SES respondents reported that prescribed medication helped them compared to the moderate-high SES group. There were no associations between the helpfulness of any method and gender, quit duration, or education.

**Table 2:** Recent quitters' perceived helpfulness of cessation methods by socio-demographic and quitting characteristics

	PERCEIVED HELPFULNESS OF METHODS (%)												
	Aided methods										Unaided methods (n)		
	
	NRT	Dentist	GP	Pharmacist	Health prof.	Natural therapy	Prescribed meds	Quitline	Self-help materials	Online	Change to light	Cut down	Cold turkey
**Gender**													
Male	92.5	62.5	87.9	76.5	92.5	60.0	96.8	64.5	76.5	100.0	62.5	90.1	95.0
Female	88.3	84.2	85.2	91.9	80.6	66.7	87.5	67.3	87.0	86.7	65.0	87.7	94.0
**Age (yrs)**													
18-30	80.0*	100.0	70.6*	72.7	71.4	85.7	93.3	88.9^a^	95.5*	100**	84.0*	93.2	96.1
31-50	90.9	79.2	87.9	86.1	86.2	68.8	92.9	72.1	85.4	95.7	62.5	88.3	93.4
51+	92.7	57.1	89.1	88.0	90.0	53.3	90.3	54.8	68.0	62.5	51.4	86.7	94.7
**Education**													
Low	92.5	80.0	87.6	91.2	84.8	50.0	89.3	72.2	87.9	81.3	67.4	87.9	93.3
High	88.2	68.2	85.0	77.8	87.8	70.2	93.0	63.0	80.0	96.3	60.0	89.5	95.3
**SES**													
Mod-high	89.9	75.0	86.1	83.8	83.3	62.8	83.8**	61.1	78.5	89.7	65.2	86.4	95.2
Low	90.4	66.7	86.9	86.7	92.6	69.6	98.4	82.1	95.2	90.0	57.9	92.4	93.2
**Quit duration**													
0-6 mths	89.4	71.0	85.6	86.0	90.7	60.5	91.1	63.9	82.8	85.7	65.2	87.9	93.6
7-12 mths	91.5	81.8	87.7	78.6	81.8	70.4	90.6	77.3	83.9	100.0	61.5	90.6	95.8

Note. Health prof = Health professional advice; Edu = Education (low = less than Year 12, high = some or completed tertiary); SES = socio-economic status; statistical tests of differences between groups are chi-square or Fisher's Exact test; **p < .01, *p < 0.05; ^a^chi-square test not significant but linear trend *p *< 0.05.

### Multivariate Associations between Characteristics of Recent Quitters and Use and Helpfulness of Cessation Methods

Backwards step-wise logistic regression analyses were conducted to predict the use of cessation methods which fell into Quadrants I, II or III and had substantial sample sizes; namely, quitting cold turkey, using NRT, receiving GP advice, gradual reduction, and using prescribed medication (see Table 3). The model predicting gradual reduction showed no significant predictors, and is therefore not shown.

**Table 3:** Logistic models predicting recent quitters' use of cold turkey, NRT, GP advice, and prescribed medication

Outcome	Predictor	OR (95% CI)
**Cold turkey**	**Age (yrs)**	
(*n *= 1028)	51+	1
	31-50	1.18 (0.87-1.59)
	18-30	2.28 (1.55-3.35)
	**SES**	
	Low	1
	Mod-high	1.42 (1.08-1.87)
	**Quit duration**	
	0-6 mths	1
	7-12 mths	1.66 (1.24-2.22)

**NRT**	**Age (yrs)**	
(*n *= 1067)	18-30	1
	31-50	2.26 (1.55-3.30)
	51+	2.33 (1.58-3.44)
	**Quit duration**	
	7-12 mths	1
	0-6 mths	1.39 (1.04-1.85)

**GP advice**	**Age (yrs)**	
(*n *= 1054)	18-30	1
	31-50	1.99 (1.30-3.06)
	51+	3.21 (2.10-4.91)
	**Education**	
	High	1
	Low	1.56 (1.16-2.09)
	**Quit duration**	
	7-12 mths	1
	0-6 mths	1.37 (1.003-1.86)

**Prescribed medication**	**Age (yrs)**	
(*n *= 1055)	18-30	1
	31-50	2.44 (1.34-4.43)
	51+	3.70 (2.05-6.67)
	**Education**	
	High	1
	Low	1.69 (1.16-2.47)
	**Quit duration**	
	7-12 mths	1
	0-6 mths	2.34 (1.52-3.61)

Note. OR = Odds Ratio; CI = Confidence Interval; Edu = Education (low = less than Year 12, high = some or completed tertiary); SES = socio-economic status.

In the model predicting quitting cold turkey, being younger in age, having a longer quit duration, or being moderate-high SES were significant predictors. For NRT, recent quitters who were either older in age or had a shorter quit duration were more likely to report using NRT than their respective reference groups. In the model predicting use of GP advice, being less educated, older in age, or having a shorter quit duration were significant predictors. For use of prescribed medication, education, age, and quit duration were retained in the final model. This model had the same set of predictors as the model for GP advice.

When considering multivariate predictors of perceived helpfulness of the methods, only prescribed medication had more than one potential predictor (*p *< .02). In the logistic regression analysis to predict perceived helpfulness of prescribed medication, gender and SES were retained in the final model, with male quitters marginally more likely to find prescribed medication helpful than females (OR = 4.18, 95% CI[0.94-18.55], *p *= 0.06) and low SES quitters more likely to find it helpful than moderate-high SES quitters (OR = 13.17, 95% CI[1.57-110.47], *p *= 0.02).

## Discussion

Consistent with a growing body of literature documenting that the majority of ex-smokers successfully quit without assistance, in the current study, more than two-thirds of recent quitters reported that they had used the cold turkey method to help them quit. Because this study did not explicitly ask about cessation methods used in their last quit attempt, we cannot claim that these smokers finally quit successfully using cold turkey. Nonetheless, our measure of perceived helpfulness for each of the methods provides a good indication that not only is cold turkey used by a large proportion of smokers in quitting and attempting to quit, it is also perceived as being more helpful than any other method. Research shows that smokers often make more than one attempt to quit smoking [22,23], potentially using a variety of methods either at the same time or over the course of many quit attempts [4]. In this case, the measure of perceived helpfulness is particularly useful, as it allows quitters to make a distinction between the methods that have helped them and those which have not.

Pharmacological aids such as NRT and prescribed medication were also considered helpful by most of the recent quitters who used them. However, NRT was used by less than one third of recent quitters, and prescribed medications less than that. This level of use, despite widespread promotional activity, is consistent with evidence suggesting that, as a *population-based *strategy, pharmacological aids are unlikely to have as great an impact on lowering smoking prevalence rates as cold turkey [24,25].

From the quadrant analysis, it was apparent that GP advice, though used relatively frequently, was rated as just below the average level of helpfulness. In 2009, 40% of a sample of NSW smokers had discussed their smoking on their last GP visit, and even more (63%) were open to seeking advice from their doctor [4]. In combination, these findings confirm that physicians' perceptions of patients' disinterest in quitting [26] should not be a barrier to their engagement in cessation assistance.

From our analysis of individual characteristics associated with use of the various cessation methods, we can develop profile of quitters who used assisted method of quitting. We found that older quitters were more likely to have used GP advice or prescribed medication than younger quitters; less educated quitters were more likely to have used GP advice or prescribed medication than more educated; and individuals who had been quit for less than six months were more likely to have used NRT than those who had been quit for longer. Conversely, the quitters who reported quitting cold turkey were either younger, had been quit for longer, or were of moderate-high SES. Consistent with previous research [3,6,7,11], these findings suggest that more heavily addicted smokers (based quit duration and age) are more likely to use assistance in quitting, while less addicted smokers are more likely to quit cold turkey. These quitting profiles have implications for targeted promotion of assisted cessation services and products.

Emerging research has suggested that internet-based cessation programs might be effective [27], and this is supported by the relatively high perceived helpfulness of online programs in the current study. However, the overall use of online programs was low. In a recent study, only 24% of 'quit smoking' searches ended at a professional smoking cessation website, with many searches ending at alternative therapy sites [28], perhaps indicating a paucity of accessible and effective programs. Though the numbers of younger quitters who used online programs in the current study were small, those who did were more likely than older individuals to report that they were helpful. Future research might explore ways to increase young smokers' engagement with effective online support.

This study, along with others [29-31], shows a very low utilisation rate of telephone cessation helplines. In 2009, 81% of ex-smokers and smokers who had tried to quit in NSW were aware of the Quitline but had never called it [4], suggesting that factors other than awareness might influence use of this service. The low use and perceived helpfulness of the Quitline among NSW quitters suggests that, in its current form, this mode of service may not be an effective *population-level *strategy for lowering smoking prevalence. Future research might explore how to increase the helpfulness of the service through integration with online support and whether the service may be best promoted to certain subgroups of smokers. The results of this study are also in accordance with accumulating evidence that some cessation aids - such as self-help materials [32] and natural therapies [33] - have only marginal helpfulness. Interestingly, of the 105 individuals in this study who reported that 'something else' helped them quit, about 40% of their responses related to advice, support or urging from family and friends. Other researchers have noted this [3], and it supports recent research indicating that conversations with family and friends might play an important role in the quitting process, and that anti-smoking media campaigns might be helpful in stimulating these kinds of conversations[34,35].

Strengths of this study are the relatively large sample of recent quitters and the breadth of cessation methods included. However, it relied on retrospective recall of cessation methods, with the possibility that memories of quitting could have become distorted over time. We found an association between duration of quitting and use of cold turkey methods. There are several potential explanations for this. The first is that individuals who have more durable cessation are more likely to have used cold turkey methods, perhaps because they smoked occasionally and did not need more intensive cessation assistance. An alternative explanation is that the longer the duration of cessation, the more likely it is that individuals forget about other methods which may have been helpful such as NRT and instead recall their personal efforts. However, we prompted respondents with each of the different cessation methods in an effort to capture all methods used, rather than simply 'top-of-mind' recall. Our analyses indicated no association between perceived helpfulness of each reported cessation method and duration of quitting. The somewhat low response rate of this survey may have led to some unknown bias, though this rate is comparable with other population surveys which use similarly conservative estimates both in Australia [36] and the U.S. [37]. Further, the rates of use of the various cessation methods in this study were similar to that in other surveys of NSW smokers [4,8], suggesting that the sample is representative in this respect. We also note that the survey did not include questions about adherence to any treatment guidelines, details about prior cigarette consumption or quitting history.

## Conclusion

Current U.S. Clinical Practice Guidelines recommend that all smokers be advised to use medication and behavioural treatment in quitting [23]. Chapman and Mackenzie raise a potential negative consequence of all smokers being imbued with the message that serious cessation efforts require treatment: they might become disempowered and inhibited in their quit attempts through self-defeating fatalism [25]. Our study confirms that many smokers quit without pharmacological or other interventions and that methods such as quitting cold turkey or gradual reduction are as likely to be rated as helpful as assisted cessation methods. This finding highlights the opportunity for presenting this empowering message to smokers who are ready to try to quit. At the same time, we acknowledge the importance of providing effective support to smokers who need it particularly more heavily nicotine dependent individuals. In addition, population-level strategies such as smoke-free environments [38], graphic pack warnings [39], increased cigarette prices [24], and well funded and effective media campaigns [7,24], must continue to be emphasised, as they are important sources of motivation and support for smokers - particularly young adults - who make less use of conventional forms of quitting assistance.

## Competing interests

The authors declare that they have no competing interests.

## Authors' contributions

WH led the design, conducted the analysis and led the interpretation of results, and contributed to the first draft of the article. SD drafted the article. DP managed the data and oversaw the analysis. TC conceived the study. All authors aided in the interpretation of the results, as well as critically reviewed and approved the final article.

## Funding

This work was not supported by any funding.

## Pre-publication history

The pre-publication history for this paper can be accessed here:

http://www.biomedcentral.com/1471-2458/11/592/prepub
